# Epidermal growth factor receptor expression in different subtypes of oral lichenoid disease

**DOI:** 10.4317/medoral.19452

**Published:** 2014-06-01

**Authors:** Dionisio A. Cortés-Ramírez, María J. Rodríguez-Tojo, Juan C. Coca-Meneses, Xabier Marichalar-Mendia, José M. Aguirre-Urizar

**Affiliations:** 1 DDS, PhD, Oral Medicine and Oral and Maxillofacial Pathology Unit. Dental Clinic Service. Master in Oral Pathology. Department of Stomatology II. UFI 11/25. University of the Basque Country / EHU. Leioa. Spain; 2 BSc, PhD, Oral Medicine and Oral and Maxillofacial Pathology Unit. Dental Clinic Service. Master in Oral Pathology. Department of Stomatology II. UFI 11/25. University of the Basque Country / EHU. Leioa. Spain; 3 DDS, MS, Oral Medicine and Oral and Maxillofacial Pathology Unit. Dental Clinic Service. Master in Oral Pathology. Department of Stomatology II. UFI 11/25. University of the Basque Country / EHU. Leioa. Spain; 4 BSc, Oral Medicine and Oral and Maxillofacial Pathology Unit. Dental Clinic Service. Master in Oral Pathology. Department of Stomatology II. UFI 11/25. University of the Basque Country / EHU. Leioa. Spain; 5 MD, DDS, PhD, Oral Medicine and Oral and Maxillofacial Pathology Unit. Dental Clinic Service. Master in Oral Pathology. Department of Stomatology II. UFI 11/25. University of the Basque Country / EHU. Leioa. Spain

## Abstract

The oral lichenoid disease (OLD) includes different chronic inflammatory processes such as oral lichen planus (OLP) and oral lichenoid lesions (OLL), both entities with controversial diagnosis and malignant potential. Epidermal growth factor receptor (EFGR) is an important oral carcinogenesis biomarker and overexpressed in several oral potentially malignant disorders. Objectives: To analyze the EGFR expression in the OLD to find differences between OLP and OLL, and to correlate it with the main clinical and pathological features. Material and Methods: Forty-four OLD cases were studied and classified according to their clinical (Group C1: only papular lesions / Group C2: papular and other lesions) and histopathological features (Group HT: OLP-typical / Group HC: OLP-compatible) based in previous published criteria. Standard immunohistochemical identification of EGFR protein was performed. Comparative and descriptive statistical analyses were performed. Results: Thirty-five cases (79.5%) showed EGFR overexpression without significant differences between clinical and histopathological groups (*p*<0.05). Histological groups showed significant differences in the EGFR expression pattern (*p*=0.016). Conlusions: All OLD samples showed high EGFR expression. The type of clinical lesion was not related with EGFR expression; however, there are differences in the EGFR expression pattern between histological groups that may be related with a different biological profile and malignant risk.

** Key words:**Oral lichenoid disease, oral lichen planus, oral lichenoid lesion, oral carcinogenesis, EGFR.

## Introduction

Oral lichenoid disease (OLD) includes different chronic inflammatory processes with an immunological basis such as oral lichen planus (OLP) and oral lichenoid lesions (OLL) (1), both entities with controversial diagnosis and malignant potential (1-4). The clinical and histopathological links between OLD subtypes are the presence of lineal papular lesions with reticular pattern, usually associated with atrophic, erosive, ulcerative and plaque lesions; and the presence of predominantly lymphocytic chronic inflammatory infiltrate with a “band like” pattern and epithelial basal cell degeneration (1,5). Clinical and histopathological differentiation between OLP and OLL is difficult, frequently even impossible to establish (2,6). Nevertheless, it seems that this differentiation is important, since some studies have demonstrated that OLP and OLL have different malignant potential (1,7,8).

OLP malignant transformation rate has been reported to range between 0 to 5%, although it is considered not to exceed 1% (9-11). Due to the lack of strict and uniform diagnostic criteria for OLP, several studies have included indistinctively cases of OLP and OLL and even other lesions with a recognized malignant potential but neither lichenoid features nor inflammatory etiology such as leukoplakia and erithroplakia (2,3,12). Interesting studies (7,8), have demonstrated that only lesions diagnosed as OLL (based on strict clinical and histopathological diagnostic criteria) showed malignant transformation risk, suggesting that the distinction of these processes is crucial for prognosis and treatment (2). Therefore, finding molecular differences between both processes is important. To the best of our knowledge, no studies have analyzed the immunohistochemical expression of biomarkers associated with oral carcinogenesis such as the epidermal growth factor receptor (EGFR) in OLP and OLL, using the van der Meij and van der Waal histological diagnostic criteria (2).

EGFR is a transmembrane glycoprotein member of the Erb growth factor receptor family (Erb1 o EGFR, Erb2, Erb3 y Erb4) which has been associated with oral carcinogenesis (13-17). EGFR regulates several mechanisms involved in cell development and epithelial integrity (15). The EGFR has a tyrosine-kinase dependent action structured by extracellular, transmembrane and intracellular zones. The binding of the extracellular component to its respective ligands (EGF [epidermal growth factor], TGF-α [transforming growth factor], betaceluline, amphireguline and hereguline), activates multiple intracellular stimulation and/or modulation pathways (Ras/Raf/MAPK; P13K/AKT; PCLgamma; STATs) such as: cell proliferation, differentiation, inhibition of apoptosis, angiogenesis, migration and cellular invasion (16).

Some studies have demonstrated that EGFR is overexpressed in oral squamous cell carcinoma (OSCC) (16), and associated with positive lymph nodes in patients with head and neck carcinomas (HNC) (18,19). Furthermore, other studies (13-17) have demonstrated a progressive increase of EGFR expression, which was proportional to the severity of premalignant lesions (13).

Despite the strong association of EGFR overexpression with oral carcinogenesis of oral potentially malignant lesions (OPML), few studies have analyzed its expression in OLP (20,21), showing controversial results, and none in OLL. On one hand, Ebrahimi *et al*. (20) have observed low EGFR expression in OLP samples, in contrast, Kumagai *et al*. (21) observed a high expression in all their samples of OLP.

Encouraged by the interesting and reproducible results obtained by van der Meij and van der Waal (7,8) and the strong association of EGFR expression and oral carcinogenesis, the aim of our study is to analyze the differences in EGFR expression in OLD subtypes such as OLP and OLL when diagnostic histological criteria are employed; and also to correlate EGFR expression with the main clinical and histological features.

## Material and Methods

We have studied 44 biopsies obtained from patients clinically and histopathologically diagnosed with OLD in the Oral Medicine and Oral and Maxillofacial Pathology Unit (Dental Clinic Service of the University of Basque Country/EHU), during the period of January 2006 to June 2008. Out of 44 patients, 30 (68.2%) were females and 14 (31.8%) males, with a mean age of 56.4 years (range 31-82 years).

Clinical and histopathological data were collected using a protocol based on previous studies (2,3,12). Briefly, the clinical data like sex, age, type and site of the lesions were collected. Regarding histopathological features, presence or absence of the main epithelial and inflammatory infiltrate characteristics were recorded and graded in mild, moderate or severe in each case. Cases were clinically classified in: Group C1 with 26 (59.1%) cases with only papular reticular lesions, and Group C2 with 18 (40.9%) cases with papular reticular and other lesions such as atrophic, erosive, and ulcerative and/or plaque lesions. As inclusion criterion, none of the patients should have been receiving treatment for OLD at the time of the study or previous to diagnosis. The mean follow up time was 43.5 months (range 20-78 months), period in which no malignant transformation was observed in any of the cases.

- Histology and Immunohistochemistry 

The cases were classified histopathologically following the diagnostic criteria proposed by van der Meij and van der Waal (2) in an “histologically typical of OLP” (Group HT) with 23 (52.3%) cases, and “histologically compatible with OLP” (Group HC) with 21 (47.7%) cases. Briefly, HT cases had to show: (1) well-defined superficial “band like” predominantly lymphocytic chronic inflammatory infiltrate, (2) epithelial basal cell degeneration, and (3) absence of dysplasia; and HC cases did not show either one or none of the characteristics described for HT cases. Cases with epithelial dysplasia were excluded from the study. Samples of oral mucosa without epithelial and/or inflammatory alterations and OSCC were employed as controls. All cases and controls underwent an hematoxylin and eosin standard histological procedure as well as conventional immunohistochemical analysis.

For immunohistochemical analysis, 4ųm paraffin sections were treated with a citrate buffer solution at 100°C for 2 minutes as antigen retrieval and incubated using the Novolink Polymer Detection Kit (Novocastra®, New Castle Upon Tyne, UK) following the manufacturer´s instructions. All cases were incubated with the primary prediluted monoclonal antibody anti-EGFR protein (clon 31G7, Zymed Labs®, Camarillo CA, USA.) for 1 hour at room temperature. The antibody employed has been tested previously in other studies (18,22). For visualization, sections were colored with the substrate/chromogen 3,3= -diaminobenzidine (DAB) using the Polymer Detection Kit (Novocastra®, New Castle Upon Tyne, UK) showing a visible brown precipitate at the antigen site. Based on previous studies (23), and considering the EGFR expression in controls, a semiquantitative analysis of the expression percentage and the expression pattern of epithelial cells was performed, using the Soft Image System Cell A software (Olympus®, Munster, Germany). Immunohistochemical analysis was performed randomly in 5 fields by 3 observers (DC, JCC and JMA), independently and without knowledge of clinical data, using a light optic microscope Olympus® BX41 20x objective and 10x ocular. A consensus agreement was reached for any given sample when discrepancies existed among observers for any given sample. The epithelial EGFR cell expression was assessed as follows: mild expression when <20% of epithelial cells were positive, moderate expression when ≥20% but <40% of epithelial cell were positive and severe expression when ≥40% of cells were positive. For statistical analysis, the variables were dichotomized in “low EGFR expression” when the expression was mild or moderate, and “overexpression of EGFR” when it was severe. The expression pattern was assessed in: membrane pattern (Mm), cytoplasmatic pattern (Ct) and mixed membrane-cytoplasmatic pattern (Mm-Ct), and the expression intensity in mild, moderate and severe. For statistical analysis the cellular expression was dichotomized in mild and moderate-severe intensity.

The study was approved by the Ethics, Investigation and Teaching Committee (CEISH) of the University of the Basque Country/EHU. The data underwent a descriptive and comparative statistical analysis with X2 Pearson method and Fisher exact test, using the statistical software SPSS (Version 15.0, SPSS Inc., Chicago, IL).

## Results

**Figure 1:**
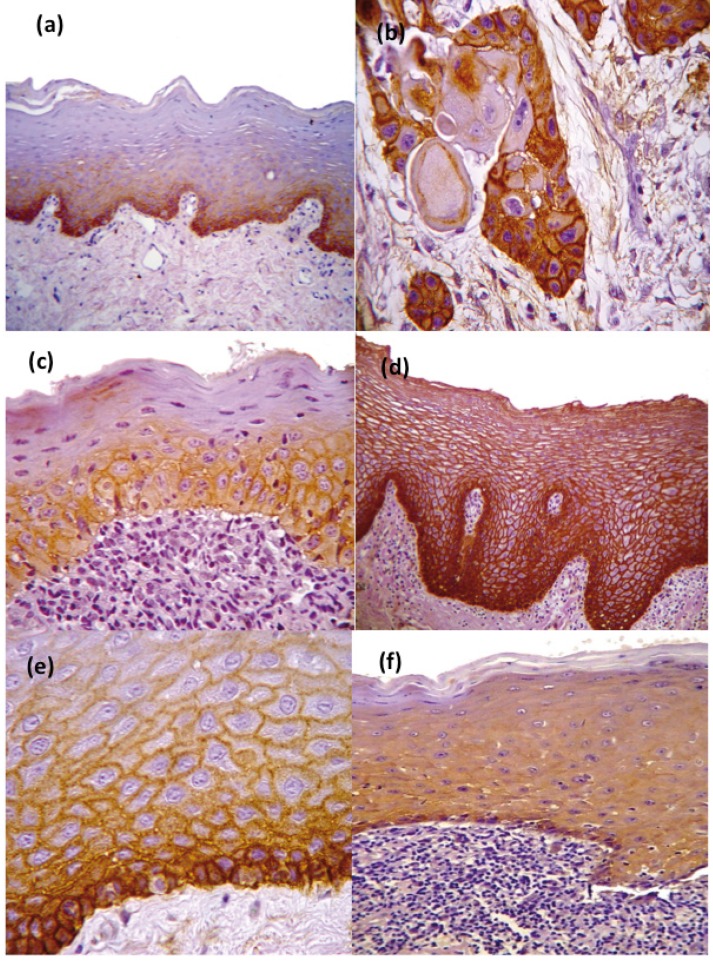
EGFR expression of not altered oral mucosa (a) (20x) and OSCC (b) (40x) controls. Mild EGFR cytoplasmatic expression pattern in HT case (c) (40x) and moderate-severe in HC case (d) (20x). Example of membrane (e) (40x) and cytoplasmatic expression pattern (f).

As expected, given the location of proliferating cells, the epithelial expression of EGFR in controls of non-affected oral mucosa was confined to basal and parabasal cell layers with an Mm expression and scarce Ct expression pattern. In contrast, OSCC controls showed an intense EGFR expression in the periphery and the center of neoplastic nests, with a mixed Mm-Ct severe expression pattern (Fig. 1).

All cases showed EGFR expression in basal and parabasal epithelial cells. Thirty-nine cases (88.6%) showed EGFR expression in the spinous layer and only in 5 (11.4%) cases reached the superficial layers. EGFR overexpression was recognized in 35 (79.5%) cases and low expression in 9 (20.5%) (Fig. 1). We think that chronic inflammation mediators might be the main reason of this high number of cases with EGFR overexpression, rather than a higher malignant risk. All cases showed an Mm expression pattern, but in 31 (70.5%), we also observed a Ct pattern, which was mild and moderate-severe in 13 (41.9%) and 18 (58.1%) cases respectively. This was an interesting finding because main EGFR expression pattern in non-affected oral mucosa controls was Mm rather than Ct, which in turn was intense in OSCC controls.

We did not find significant differences in EGFR expression between the clinical and histological groups (*p*>0.05) ( Table 1), suggesting that the expression of EGFR in OLD subtypes does not seem to be completely related with the severity of the clinical lesions, in other words, epithelial integrity (ulcers and/or erosions) did not affect significantly EGFR expression. Out of the cases with EGFR overexpression, 17 (38.6%) cases showed a moderate-severe Ct expression pattern (Fig. 1), 6 (40%) and 11 (84.6%) cases from HT and HC groups respectively showing a significant difference (*p*=0.016) ( Table 1). This finding suggests, that when specific histological diagnostic criteria (2) are applied there are differences in the cellular localization of EGFR in OLD subtypes, regardless of other histological features like inflammatory infiltrate intensity and/or epithelial thickness or integrity.

**Table 1:**
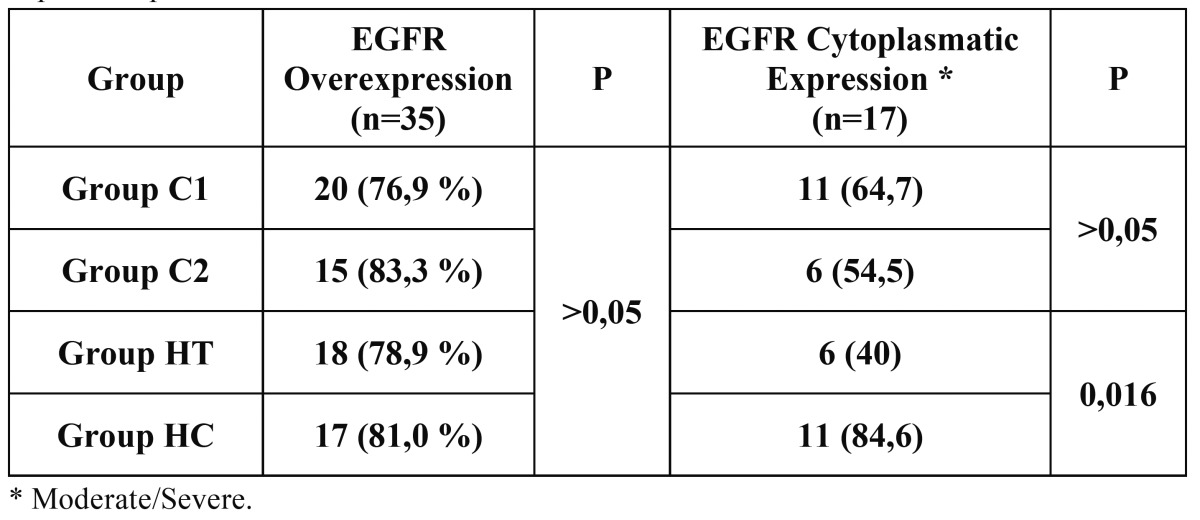
Clinical and histological cases of OLD with EGFR overexpression and cytoplasmatic expression pattern.

When we analyzed the histopathological features of cases with EGFR overexpression, we only observed significant differences regarding the keratinization type, since 19 (54.3%) of these cases showed parakeratosis compared with only 1 (11.1%) case without EGFR overexpression (*p*=0.027) ( Table 2). To this respect, we thought that is difficult to consider this association, since parakeratosis is frequently observed in OLP and OLL samples. However, it might be reasonable to observe modifications in the epithelial lining features such as keratosis, hyperkeratosis, atrophy or basal cell degeneration; or from the inflammatory infiltrate such as the intensity, cellular types and pattern with different EGFR expression intensity. This suggests that EGFR expression intensity not always has a histological visible effect in OLD subtypes.

**Table 2:**
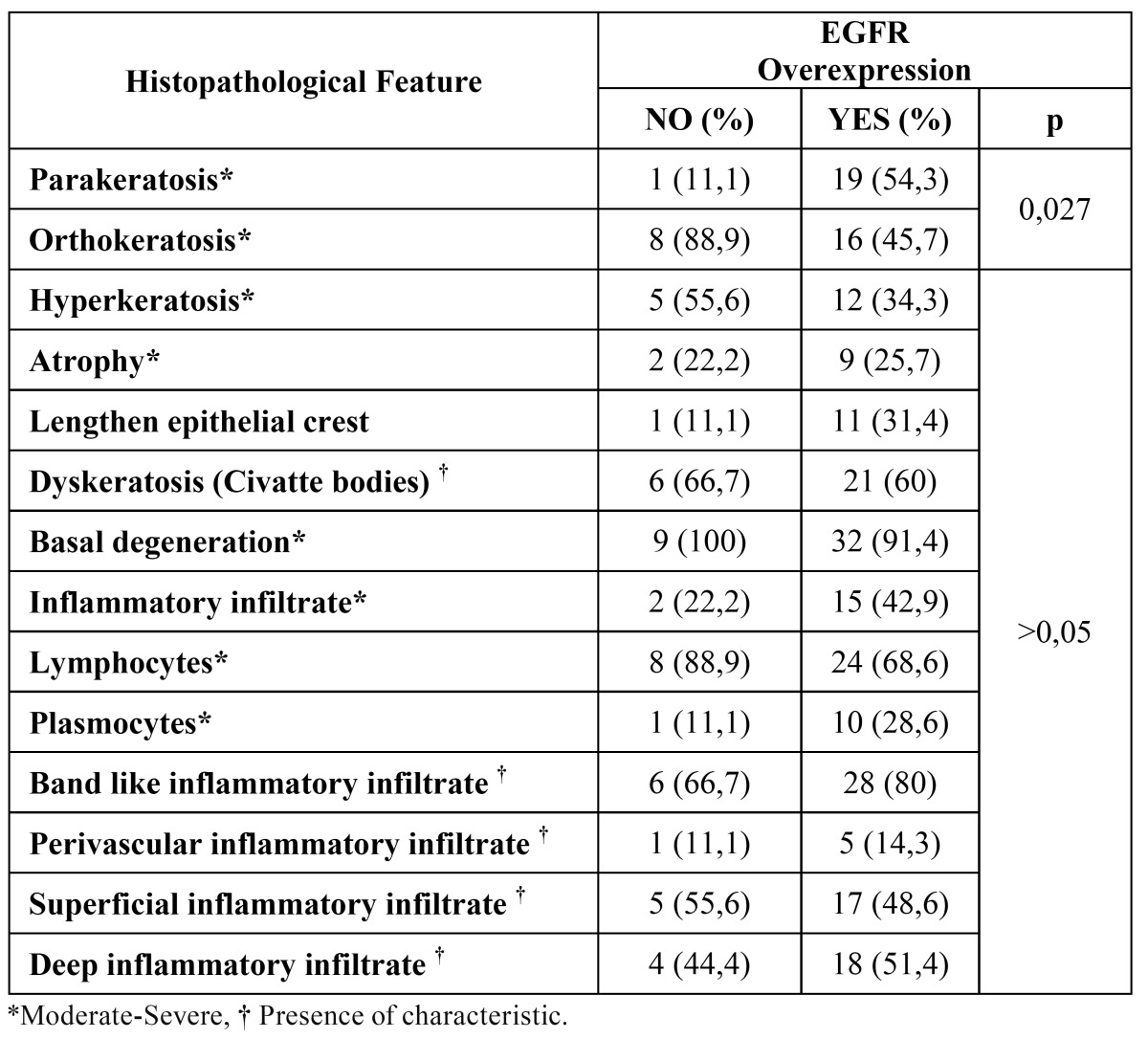
EGFR overexpression according to the main histopathological features observed.

## Discussion

OLD includes different chronic inflammatory processes like OLP and OLL, characterized clinically by the presence of white lineal papular lesions in the oral mucosa (1). The malignant potential of these processes is controversial and sometimes difficult to analyze due to the lack of defined and uniform diagnostic criteria employed in each study (7-10). Several years ago, van der Meij and van der Waal (2) proposed a clinical and histological diagnostic criteria to differentiate typical cases of OLP from those that were only compatible called OLL. These authors (2) demonstrated that when these criteria were applied, only cases diagnosed as OLL showed an evident risk for malignant transformation (7,8). Although a clear clinical and histopathological differentiation of OLP and OLL is critical, an immunohistochemical analysis of these processes would give valuable information about their molecular profiles and their possible malignant risk.

The EGFR protein is a biomarker of early carcinogenesis (24,25), overexpressed in several oral premalignant diseases (13,14,16). Moreover, EGFR has an synergic participation with other carcinogenesis biomarkers like cyclooxygenase-2 (COX-2), inducible nitric oxide synthase (iNOS) (26-28). iNOS is associated with EGFR through the stimulation of STAT-3 (Signal Transducer and Activator of Transcription-3) and with COX-2 through the synthesis of prostaglandin (PGE2) (16). The upregulation of STAT-3 and COX-2 expression maintain directly and/or indirectly the EGFR intracellular pathways, due to activation of other proteins involved in this process, enhancing oral carcinogenesis in OPML such as OLP and OLL (16,24). To this respect, previously we have demonstrated the overexpression of COX-2 in these same samples (27). These results may support the synergy between EGFR and COX-2 and highlights the possible prognostic implications of this molecular interaction in OLP and OLL.

Our study has demonstrated that EGFR is overexpressed in a great percentage of OLD cases, showing no differences between the clinical and histological groups. These findings may suggest that the clinical type of lesions does not always reflect the molecular features of OLD processes, in other words, the presence of an erosive or ulcerative lesion “clinically more aggressive and striking”, not necessarily implies an overexpression of EGFR, and in the same way, the presence of reticular white lesion “clinically less aggressive or striking“, does not necessarily imply a low EGFR expression. It is worth mentioning that these findings were also observed in a previous study (27) when we analyze the COX-2 expression in these same cases. The histological HT and HC groups showed no significant differences in the EGFR overexpression, suggesting that the presence or absence of a particular histological feature like an intense inflammatory infiltrate and/or extensive epithelial basal cell degeneration or thickness it is not related with EGFR expression in OLP and OLL. These data indicate the existence of other molecular mechanisms implicated in EGFR expression in OLD subtypes.

Due to the lack of clinical and histological separation in similar studies (20,21), the comparison of our results is difficult. However, our results contrast with those obtained by Ebrahimi *et al*. (20) where, although they do not mention the type of clinical lesion of their cases, they observed a low EGFR expression in OLP samples compared with controls. These authors (20) suggest that high p53 expression in OLP or the presence of mutated EGFR protein (EGFR vIII) may explain the low EGFR expression in OLP. p53 protein is one of several control mechanisms of EGFR expression (24,25), thus its overexpression may explain EGFR low expression. However, several studies (13,17,29) have pointed out that EGFR mutations in OLP and other OPML is an uncommon event. Moreover, several studies (16,25,29) have demonstrated that the expression of EGFR vIII is mainly observed in late oral carcinogenesis stages and always accompanied with the expression of the wild type EGFR protein. In this sense, the antibody used in this study recognizes both wild and mutated EGFR protein, which makes it difficult to ensure the main type of EFGR protein detected in our study. However, based on previous studies (13,17,29) and considering the low percentage of cases with expression of EGFR vIII in OLD and other OPML, we assume that the main protein detected in our study is the wild type EGFR protein. Similar to our results, Kumagai *et al*. (21) observed EGFR overexpression in all OLP samples, and additionally they observed overexpression of EGFR ligands (amphireguline, epireguline y HB-EGF [Heparin-binding-EGF like growth factor]) and decreased expression of the ligands EGF and TGF-alpha. Supported by their results, these authors (21) suggest that EGFR overexpression in OLP may contribute to the carcinogenesis of this disorder. These authors (21) have only included reticular lesions, therefore the comparison with our results is not totally valid in this sense. However, we did not observe significant differences in EGFR expression among the Group C1 (papular-reticular) cases.

Most of our samples showed a mixed Mm-Ct EGFR expression pattern which is in concordance with other studies (13,21). However, we observed significant differences between the histological groups, since high percentage of HC cases showed a Ct moderate-severe expression pattern compared with HT cases. Other studies (20,21) that analyze the EGFR expression in OLP make no reference to this finding. It is worth to note that this Ct expression pattern was not present in controls of non-affected oral mucosa but was intense in OSCC controls. To this respect, Muller *et al*. (18) have linked previously the Ct EGFR expression pattern with malignant potential in patients with HNC. These authors (18) observed that Ct EGFR expression pattern in HNC cell lines was associated with nodal metastasis and increased resistance to tyrosine-kinase inhibitors. Other studies (13), have demonstrated in samples of normal oral mucosa adjacent to HNC, that the localization of Ct EGFR expression and the ligand TGF-alpha was the same, and furthermore, the intensity and extension of this expression increased proportionally to the severity of the lesion. Similarly, Srinivasan *et al*. (17) have observed that Ct EGFR expression in OPML proportionally increased with the severity of the epithelial dysplasia. These authors suggest (17) that the Ct EGFR expression could be due to the internalization of the receptor once it has been activated by extracellular ligands, or alternatively, due to it being transport to its final location after being synthesized. If the presence of an increased Ct EGFR expression in HT or OLL cases is associated or may contribute to a greater malignant potential as was demonstrated previously by van der Meij *et al*. (7,8) is difficult to ascertain with these results, but may be worth considering.

We have demonstrated that EGFR overexpression was not associated with any particular clinical or histological feature, suggesting that different and/or more complex molecular mechanisms are involved in this process. The main cause of EFGR overexpression in OLD samples remains unknown. However, some authors (22) have found that EGFR overexpression in other OPML with malignant transformation was associated with an increased EGFR gen copy number, which can also be a possibility that need to be investigated in OLD subtypes.

Considering the low rate of malignant transformation of OLP (<1%) (9,11), it is obvious that the high percentage of cases with EGFR overexpression herein observed will not suffer a malignant transformation, since several genetic alterations would be necessary to make that possible. Nevertheless, the EGFR and COX-2 overexpression previously demonstrated (27) in these samples, may enhance the carcinogenic process in those genetically susceptible cases. Therefore, these molecular alterations can give us valuable information about intracellular pathways altered in OLD subtypes like OLP and OLL. So, with all the previous background, we can propose two possible scenarios: the first that HC subtypes lesions with EGFR overexpression and increased Ct expression pattern may have a different biological behavior from those HT subtypes lesions, which might be possibly associated with a higher malignant potential; the second scenario, may be that these differences in the EGFR expression pattern, could be the result of different molecular profiles involved directly or indirectly in EGFR immunoexpression in OLP and OLL, which is probably determined by etiological factors of each process and may not be related with malignant potential. However, regardless the prognostic implication of these immunohistochemical differences, we have demonstrated that although the clinical and histopathological features are very similar in all OLD processes, there are molecular differences in each OLD subtype.

We did not observe significant association between EGFR expression and any particular histological feature, except for keratinization type, since more than 50% of the cases with EGFR overexpression showed parakeratosis. This finding has not been reported previously by other authors (20,21). We do not know exactly the meaning of this finding, or even if it has clinical relevance. However, considering that parakeratosis is a common finding in OLD samples it is very difficult to evaluate this association. Nevertheless, what we do consider relevant is the lack of association of EGFR overexpression with the presence and/or intensity of some particular features from the epithelial lining and inflammatory infiltrate. Inflammatory intensity and epithelial thickness are some of the features classically associated with the activity of OLD lesions. We believe that these findings may have relevance in those cases with mild histological activity at diagnosis and then develop malignant transformation in a short period of time.

We can conclude that there is a high EGFR overexpression in OLD subtypes, which increase their susceptibility to the EGFR stimulation effects like cell proliferation, differentiation, apoptosis inhibition, angiogenesis, migration and cellular invasion. Neither the clinical type nor particular histological features were associated with EGFR overexpression. Cases considered as “histologically compatible” (HC) or OLL show a greater cytoplasmic EGFR expression, suggesting biological differences with HT cases. The long term follow up of these OLD patients will give us more consistent information about the significance of EGFR expression and malignant potential in these processes. However, further studies are needed to ascertain the exact prognostic value of EGFR expression in different types of OPML like OLP and OLL.
